# B7-H3 participates in human salivary gland epithelial cells apoptosis through NF-κB pathway in primary Sjögren’s syndrome

**DOI:** 10.1186/s12967-019-2017-x

**Published:** 2019-08-14

**Authors:** Ping Li, Ying Yang, Yi Jin, Rui Zhao, Chen Dong, Wenjie Zheng, Tianyi Zhang, Jing Li, Zhifeng Gu

**Affiliations:** 1 Research Center of Clinical Medicine, Affiliated Hospital of Nantong University, 20th Xisi Road, Nantong, 226001 Jiangsu People’s Republic of China; 2 Department of Rheumatology, Affiliated Hospital of Nantong University, Nantong, 226001 Jiangsu People’s Republic of China; 3 Key Laboratory of Neuroregeneration, Nantong University, Nantong, 226001 Jiangsu People’s Republic of China

**Keywords:** Primary Sjögren’s syndrome, B7-H3, p65

## Abstract

**Background:**

Primary Sjögren’s syndrome (pSS) is an autoimmune disorder mainly characterized by exocrine gland injury. Costimulatory molecules play an important role in immune-regulatory networks. Although B7 family costimulatory molecules were previously discovered in human salivary gland epithelial (HSGE) cells in pSS, the effects of the B7 family member B7-H3 (CD276) have not been well elucidated. Thus, this study aimed to investigate the role and mechanism of B7-H3 in HSGE cells in pSS.

**Methods:**

The expression of B7-H3, B7-H1, PD-1 in serum, saliva and salivary gland were examined by immunohistochemistry (IHC) and enzyme-linked immunosorbent assay (ELISA). Immunofluorescence was used to test the expression and distribution of B7-H3, AQP5 and CK-8 in salivary gland tissues. Flow cytometry, Cell Counting Kit 8 (CCK-8) and western blot (WB) were performed to research the apoptotic, proliferative and inflammatory effects of B7-H3 in primary HSGE cells and HSGE cell lines.

**Results:**

Our results showed that the expression of PD-1, B7-H1 and B7-H3 in peripheral blood, and salivary glands in pSS patients was higher than that in healthy controls, which was positive correlation with the grade of the salivary glands. The expression of B7-H3 in saliva was higher in pSS patients than that in healthy controls, which was detected with the most significant difference of them. The expression of B7-H3 in primary HSGE cells of pSS patients was significantly higher than healthy controls. B7-H3 increased activity of NF-κB pathway and promoted inflammation of HSGE cells, decreasing the expression of AQP5. Furthermore, B7-H3 overexpression inhibited proliferation and induced apoptosis in HSGE cell lines.

**Conclusion:**

B7-H3 could promote inflammation and induce apoptosis of HSGE cells by activating NF-κB pathway, which might be a promising therapeutic target for pSS.

## Introduction

pSS is a systemic autoimmune disease featured by infiltration of periductal lymphocytes in saliva and lacrimal glands, with decreased secretory function and dry mouth and eyes [1]. In the previous study, we demonstrated that pSS was correlated with the patients’ quality of life [2, 3]. Due to the extensive involvement of various epithelial cells, pSS has also been depicted as autoimmune epithelitis disease [4, 5]. HSGE cells can present antigen and induce T cell activation in pSS immunological salivary gland lesions [6–8]. Activation of T cells requires T cell receptor (TCR) and antigen peptide-MHC as the first signal and costimulatory molecules as the second signal required. The recognition of the costimulatory molecules could act as an efficient target-therapy strategy of autoimmune diseases [9–11].

According to recent reports, many costimulatory molecules of the B7 family are involved in the regulation of immune responses [12–14]. The programmed death ligand 1 (PD-L1/B7-H1), which belongs to the B7 superfamily member, is significantly expressed in immune cells. Programmed death 1 (PD-1), the member of CD28 family, is expressed on the surface of natural killer cells, dendritic cells, B cells, and T cells [15]. PD-1 signal abnormalities were related to multiple sclerosis, rheumatoid arthritis (RA) and systemic lupus erythematosus (SLE), which were important subjects of immunological research [16]. Another study revealed that the binding of B7-H1 in HSGE cells to receptor of T lymphocytes is associated with inflammation in pSS patients [17]. B7-H3, belonging to the B7 superfamily, is also known as a transmembrane glycoprotein. Earlier studies demonstrated that upregulation of B7-H3 offered an indicator of more severe activity in RA, and might participate in disease progression through inflammatory cytokine secretion, such as TNF-α [18, 19]. However, the mechanism of B7-H1/B7-H3 has not been clearly elucidated in pSS. Given that costimulatory molecules expression had not been fully characterized in pSS, we considered to make further research. First, we compared the expression of B7-H3, PD-1, B7-H1 in serum, saliva and salivary glands of healthy controls and pSS patients. After that, by operating cytological research, we found that B7-H3 increase the NF-κB pathway activity, promote inflammation and induce apoptosis of HSGE cells, resulting in lower expression of AQP5. This study indicate that B7-H3 could act as a new therapeutic target for pSS.

## Materials and methods

### Patients

99 pSS patients were collected from Affiliated Hospital of Nantong University from September 2017 to September 2018. 68 healthy controls were enrolled from the medical center population. Samples derived from healthy controls and pSS patients were matched with gender, race and age. The patients’ pSS classification was based on the 2016 American-European Consensus Group (AECG) SS classification criteria [20]. Salivary gland biopsy specimens were obtained from all participants to assess the pathological state of pSS. Donors carrying inflammatory disease, infectious disease, or tumor were excluded from the sample groups. This study was performed by accordance with the medical ethics of the Affiliated Hospital of Nantong University, with all participants signed an informed consent. Patient characteristics are presented in Table 1.

**Table 1:** Characteristics with pSS patients and healthy controls

Characteristics	pSS patient n = 99	Healthy n = 68
Age (years)	49 (46–55)	46 (40.5–63)
Gender (male/female)	2/97	4/64
Disease duration (years)	0.5 (0–3)	0
ESSPRI	3 (2–4)	NA
ESSDAI	4 (0–7)	NA
Number of caries	4 (1–8)	0
Autoantibody positivity		
Anti-SSA and/or anti-SSB	99	0
Rheumatoid factor (≥ 20)	2/3	NA
ESR (mm/h)	14 (0–24)	NA
C3 mg%	0.77 (0.61–0.97)	NA
C4 mg%	0.15 (0.13–0.22)	NA

Data are expressed as median (interquartile range) for continuous variables and as number for categorical variables

*pSS* primary Sjögren’s syndrome, *ESR* erythrocyte sedimentation rate, *ESSDAI* EULAR primary Sjögren’s syndrome disease activity, *ESSPRI* EULAR primary Sjögren’s syndrome patient-reported indexes, *C3* complement 3, *C4* complement 4, *NA* not applicable

### Saliva collection

We collected unstimulated whole saliva (UWS) using the drooling method. Patients were not allowed to stimulate the salivary flow for 90 min before saliva was obtained, i.e., by drinking, chewing, tooth brushing, use of mouthwash and smoking. At the beginning, the subjects were told that any saliva were prohibited to swallow during the collection process. The subjects were required to allow the saliva to accumulate on the floor of the mouth until enough saliva has pooled. They can tilt their head forward and let the saliva drip into the funnel collection. The subjects were allowed to gather the saliva for 5 min after it was drained into a pre-weighed cup. Saliva collections were performed between 9 and 12 a.m. to minimize the impact of circadian fluctuations during the day. Whole saliva samples were centrifuged at 10,000 rpm for 1 min at 4 °C to remove debris and cells [21]. Until time of analysis, the resulting supernatants were stored at − 80 °C. Salivary flow rates were calculated by dividing the weight of the collected saliva (grams) by the collecting time (minutes).

### ELlSA

The levels of B7-H3, B7-H1, PD-1 in saliva and peripheral blood were quantified with the ELISA kit (BSBIO, China). Briefly, the serum samples and saliva samples were seeded on 96-well ELISA plates, and incubated at room temperature for 60 min. The samples were incubated with the primary antibody for 60 min at room temperature, then incubated with HRP-conjugated secondary antibody for 60 min at room temperature. Thereafter, they were incubated with the substrate solution at room temperature for 15 min, followed by adding a stop solution. Subsequently, the absorbance was measured by a microplate reader at 450 nm.

### Immunohistochemistry analysis

We cut salivary gland tissues into 3–5 μm slices for IHC staining. We stained PD-1, B7-H3, and PD-L1 antibodies by IHC, then degreased the slices in turpentine oil and rehydrated by a series of fractional ethanol. The endogenous peroxidase was hatched by adding 3% hydrogen peroxide to the slide. Normal goat serum (ZSGB-BIO, China) blocked nonspecific protein binding for more than 1 h. The slices were incubated with monoclonal antibody PD-L1 (ab205921, Abcam, UK, 1:600), B7-H3 (ab246794, Abcam, UK, 1:100), PD-1 (ab214421, Abcam, UK, 1:1000) at 4 °C (overnight). Primary antibody was conjugated with biotinylated goat anti-rabbit IgG (Vector Laboratories, USA) at room temperature for 1 h. After all incubation and blocking steps, we washed the samples with PBS for 5 min. We stained the samples with diaminobenzidine and viewed sectioned under the microscope.

### Immunofluorescence analysis

Salivary gland tissue containing 4% paraformaldehyde was kept on ice for 2–3 h, then was left in 30% sucrose overnight at 4 °C, embedded in OCT, and sliced it with the thickness of 5–6 μm. We blocked the sections with 10% normal goat serum at room temperature for 1 h and incubated with primary antibody CK-8 (ab53280, Abcam, UK, 1:20), B7-H3 (ab246794, Abcam, UK, 1:100), AQP5 (ab78486, Abcam, UK, 1:1000) overnight at 4 °C, then incubated the second antibody goat anti-rabbit IgG (Vector Laboratories, USA) for 30 min at room temperature. Staining was developed with green fluorescent dyes (FP1494001KT, PE,1:3000), Red fluorescent dyes (FP1494001KT, PE,1:3000) and DAPI. After all incubation and closure steps, we washed the samples with PBS for 5 min. The sections were observed under the fluorescence microscope (Olympus).

### Culture of HSGE cells

#### Primary cells

HSGE cells were cultured according to the following experiment process [22]. The salivary gland tissues were obtained from healthy controls and pSS patients. In short, the tissues were cut with scalpels and fine needles and placed in six-well plates covered with type-I collagen (Iwaki, Tokyo).The medium was then added consisting 100 U/ml penicillin and 100 μg/ml streptomycin (Gibco, New York), 25 μg/ml bovine pituitary extract (Kurabo, Osaka, Japan), 0.4 μg/ml hydrocortisone (Sigma-Aldrich, MO), a limited keratinocyte-SFM medium (Invitrogen Life Technologies, CA), 10% fetal bovine serum(Gibco, New York). The tissue was incubated for several days and photographed on day 3 and day 10. When growth of HSGE cells was observed in vitro, we transferred cells to a 100-mm^2^ plate after fusion, and the plate was covered with type I collagen (Iwaki). After the HSGE cells reached junction on the 100-mm^2^ plates, the HSGE cells were cultured on small cell bottle covered with type-I collagen (Iwaki) for the next experiment. Primary cells were observed with a small number of fibroblasts which were removed by means of differential attachment technique.

#### HSGE cell lines

The reagents and materials for culture of HSGE cell lines were obtained from GIBCO BRL (Gaithersburg, USA). The experimental HSGE cell line was supplied by Shanghai Aolu Biological Technology Co.Ltd. We cultured HSGE cell line in Dulbecco’s modified Eagle’s medium, 10% fetal bovine serum, 100 U/ml penicillin, and 100 μg/ml streptomycin and maintained them in a humidified incubator containing 5% CO_2_. Cells were digested with 0.25% trypsin/EDTA solution and then washed and resuspended in new medium.

### Flow cytometry

After isolation, HSGE cells were directly stained for characteristic markers and analyzed by flow cytometry. HSGE cells were harvested by centrifugation at 300*g* for 5 min and then washed two times in PBS. We fixed cells with 4% paraformaldehyde for 1 h at 4 °C and blocked for 2 h in 0.1% Triton X-100 and 5% BSA at room temperature. The following labeled antibodies were used: CK-8 (ab53280, Abcam, UK, 1:20) antibodies at 4 °C for 30 min, followed by Alexa Fluor 488-conjugated Affinipure Donkey Anti-Rabbit IgG (H+L) (Jackson ImmunoResearch, 1:200, USA) at 4 °C for 30 min.

### Cell transfection

The cells were divided into the following four groups: the negative control group (NC: transfected with 7.5 μg lipo3000), the control group (controls: without any treatment for cells), upregulated-B7-H3 (7.5 μg B7-H3 PcDNA3.1(+) − Tat plasmid + 7.5 μg lipo3000), downregulated-B7-H3 group (7.5 μg B7-H3-shRNA + 7.5 μg lipo3000). PcDNA3.1(+) − Tat plasmid and B7-H3-shRNA were all purchased from Shanghai GenePharma Co., Ltd. (Shanghai, China). 24 h before transfection, the HSGE cell lines (3 × 10^5^/well) were plated in a six-well dish. According to the manufacturer’s instructions, the HSGE cell lines were transfected when the cell confluence reached 40–60%. In serum-free medium, Lipo3000 were diluted for transfection. The lipo3000 are mixed with plasmids and shRNA and allowed to stand for 20 min, the mixture was added into each well. The cells were cultured in serum-free medium for 6 h. After that, we replaced the medium with complete culture medium. The cell transfected after 48 h were extracted for further experiment.

### Cell proliferation assays

The proliferation of cells was measured by a CCK-8 assay (C0039, Beyotime, China). We seeded cells in 96-well culture plates at a density of 2000 cells per well, which were then incubated prior to the indicated experiments for 24 h. Cells were treated at 12, 24, 36 and 48 h. After incubation, we added CCK-8 (10 μl/well) and incubated them at 37 °C for 2 h. The optical density (OD) value of each well at 450 nm was measured using a microplate reader. The statistical differences were measured with three independent wells in each group.

### Cell apoptosis assays

The cells were detached by a trypsin solution, followed by centrifugation for washing. Cells were floated in 100 μl Annexin V Binding Buffer, 10 μl Annexin V-fluor647/PI/Apoptosis detection kit. Sequentially 5 μl propidium iodide (PI) were added for 15 min of room temperature incubation. 400 μl Annexin V Binding Buffer was added for measuring cell apoptosis on FC500 MCL flow cytometry (Beckman Coulter Inc, Fullerton, CA, USA).

### Western blot analysis

We washed harvested cells with PBS three times and then lysed cell in RIPA (Beyotime) buffer containing Phenylmethanesulfonyl fluoride (PMSF, Beyotime) and phosphatase inhibitor (Beyotime). According to the manufacturer’s protocol, we measured protein concentrations by bicinchoninic acid kits (BCA, Beyotime). By 10% and 12% SDS-PAGE electrophoresis, we separated 50-μg protein samples and then transferred them to PVDF membranes. The protein-containing PVDF membranes was blocked for 2 h with 8% skimmed milk and incubated with primary antibodies for 16 h at 4 °C. The immunoblotting: anti-AQP5 (ab78486, Abcam, UK, 1:1000), Bcl-2 (ab59348, Abcam, UK, 1:1000), Bax (ab53154, Abcam, UK, 1:1000), NF-κB p65 (phosphor S536) (ab76302, Abcam, UK, 1:10,000), cleaved-caspase3 (ab32042, Abcam, UK, 1;500), caspase3 (ab179517, Abcam, UK, 1:1000), TNF-α (ab1793, Abcam, UK, 1:1000), IL-6 (ab9324, Abcam, UK, 1;1000). PVDF membranes were rinsed with TBST buffer three times for 5 min each and then incubated with appropriate rabbit or mouse secondary horseradish peroxidase-conjugated antibodies (Sangon Biotech). After being rinsed three times by TBST (15 min each), the membranes were developed with the enhanced chemiluminescence (ECL) solution (P10300, NCM Biotech, China) at room temperature for 60 s. To quantify the expression of protein, we quantified bands by Image J software. In short, we investigated the integrated density of the samples and controlled with the standard band density (GAPDH). We set the proportion of the control group to 1 and evaluated the specified groups to decide the relative proportions of expression. The experiment was repeated at least three times.

### Statistical analysis

We compared the serological and clinical characteristics between the healthy controls and pSS patients by the Fisher’s exact test or Mann–Whitney U-test. To confirm the correlation between the costimulatory molecule in saliva and peripheral blood and the level of LSMG, we used Spearman’s rank correlation coefficient. SPSS, ver. 13 (SAS, Cary, NC) were used for all statistical analysis in this study. *P < 0.05 was considered to be indicative of statistical significance.

## Result

### Characteristics with pSS patients and healthy controls

The characteristics of the 68 healthy controls and 99 pSS patients were summarized in Table 1. Compared with the healthy controls, higher positive rates of anti-SS-A/RO antibodies were detected in the pSS patients. Besides, the pSS patients also had higher levels of ESR, C3, C4, ESSPRI, ESSDAL, the number of dental caries, and the focus score of salivary gland biopsy.

### B7-H3, PD-1, B7-H1 expression in serum, saliva and salivary gland of healthy controls and pSS patients

**Fig. 1:**
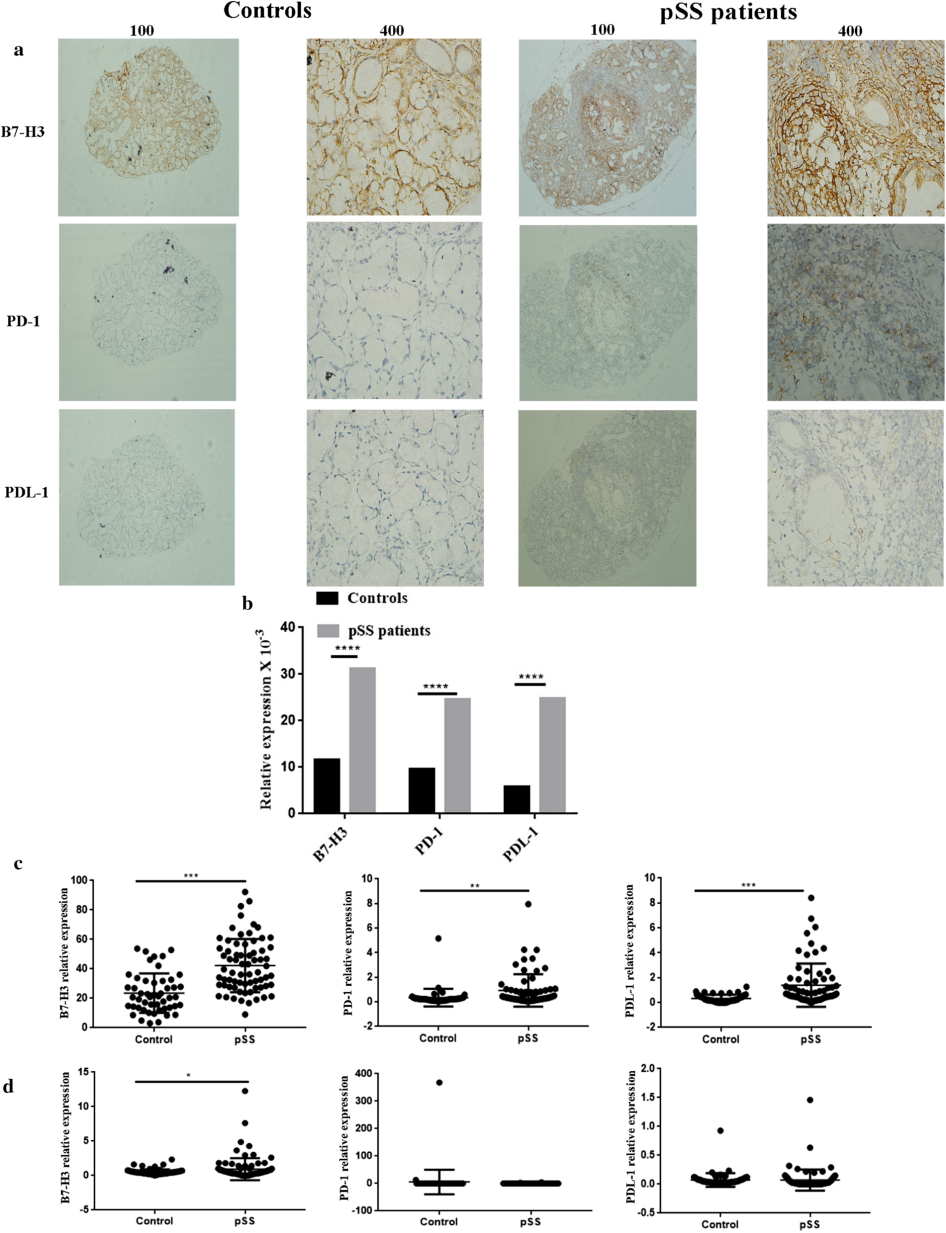
B7-H3, PD-1, B7-H1 expression in serum, saliva and salivary gland of healthy controls and pSS patients. **a** The expression of B7-H3, PD-1, B7-H1 in pSS patients was significantly higher than that in healthy controls by IHC in salivary glands tissues. **b** Quantitative analysis of the result of IHC. **c** pSS patients (n = 69) had higher serum levels of B7-H3, PD-1, B7-H1 compared to healthy controls (n = 50). **d** pSS patients (n = 99) had higher saliva levels of B7-H3 compared to healthy controls (n = 68). ***P < 0.001, **P < 0.01, *P < 0.05 vs. the healthy controls

Compared with healthy salivary gland tissues, IHC of pSS patients’ salivary gland tissues showed higher expression of B7-H3, PD-1 and B7-H1 (Fig. 1a). As expected, the stroma of normal salivary gland tissues showed diffuse brown staining, indicating the presence of B7-H3. However, there was little expression of PD-1 and B7-H1 in the salivary glands of healthy controls. In addition, epithelial cells lining the salivary gland ducts showed positive staining, indicating the presence of B7-H3. In contrast to healthy controls, the expression of B7-H3 (*P *< 0.0001) and PD-1 (*P *< 0.001), B7-H1 (*P *< 0.001) was highly positive in the stroma as well as inside the cellular profiles of the ducts in pSS patients.

The levels of B7-H3 (*P *< 0.0001), PD-1 (*P *= 0.061) and B7-H1 (*P *< 0.0001) proteins in peripheral blood tested by ELISA were elevated in pSS patients compared with healthy controls (Fig. 1b). Additionally, there was no difference in the expression of PD-1 (*P *= 0.2309) and B7-H1 (*P *= 0.9782) in saliva and an obvious difference in the expression of B7-H3 (*P *= 0.0373) between pSS patients and healthy controls (Fig. 1c).

### Difference in B7-H3, PD-1, B7-H1 proteins level between pSS patients grades I/II and III/IV

**Fig. 2:**
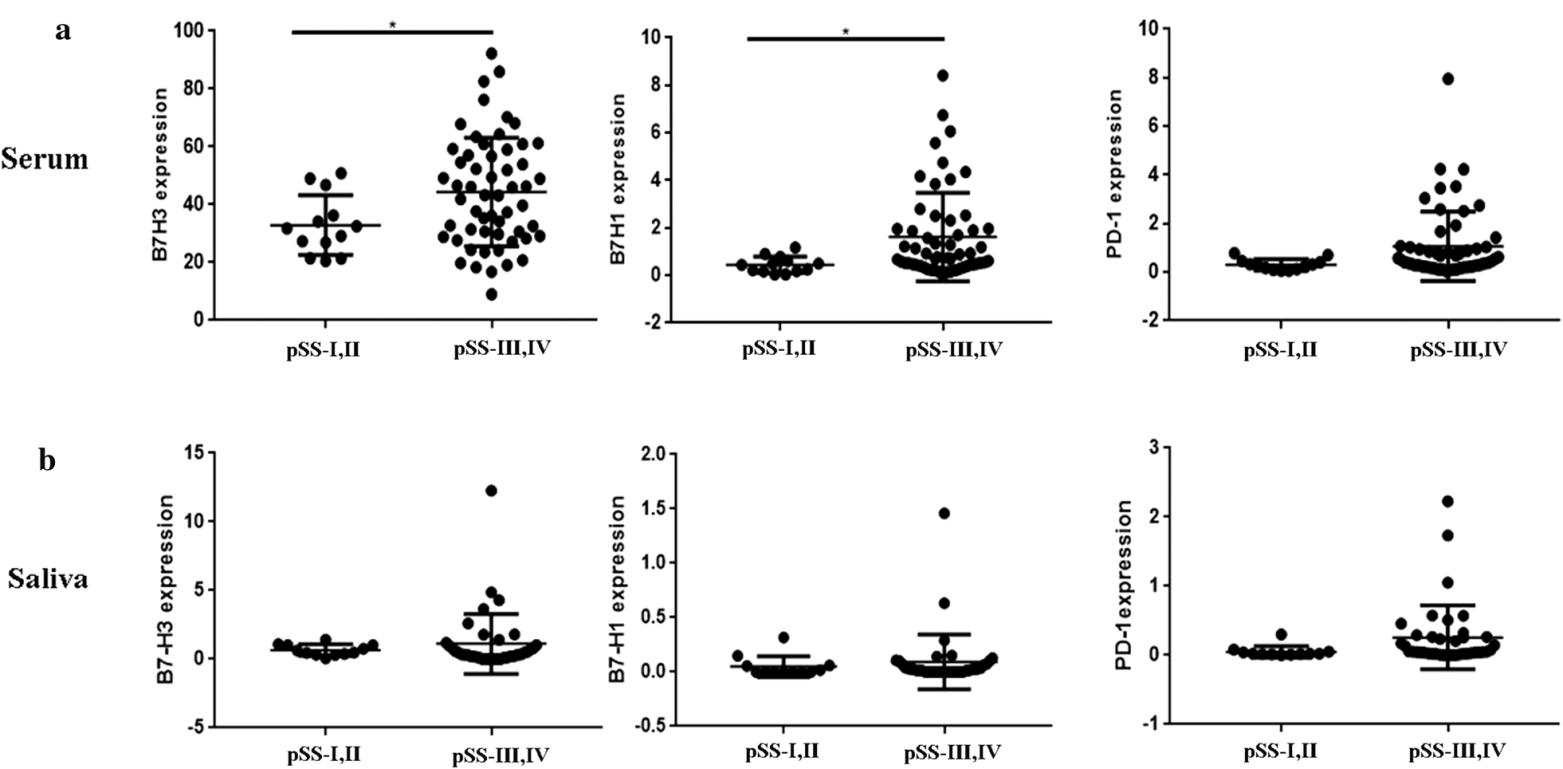
Difference in B7-H3, PD-1, B7-H1 proteins level between pSS patients grades I/II and III/IV. **a** III, IV level patients (n = 56) had higher serum levels of B7-H3, B7-H1 compared with I, II level patients (n = 13). There is no difference in the expression of PD-1 in serum between the two. **b** III, IV level patients (n = 39) had no difference in saliva levels of B7-H3, PD-1, B7-H1 compared with I, II level patients (n = 12)

To determine whether there were any differences in the expression of B7-H3, PD-1, or B7-H1 in patients with different status of salivary glands, patients were divided into two groups: I, II level patients, n = 13; III, IV level patient, n = 56. The serum levels of the B7-H3 (*P *= 0.0109), B7-H1 (*P *= 0.0305) were stronger in III, IV level patients than in I, II level patients (Fig. 2a). However, no significant differences of PD-1 (*P *= 0.0511) were found between the two groups. We also examined the expression of those molecules in saliva. In contrast, there were no significant differences in B7-H3 (*P *= 0.4789), PD-1 (*P *= 0.5633), B7-H1 (*P *= 0.1547) proteins level between I, II level patients (n = 12) and III, IV level patients (n = 39).

### The location of B7-H3 expression and the relationship between B7-H3 and AQP5

**Fig. 3:**
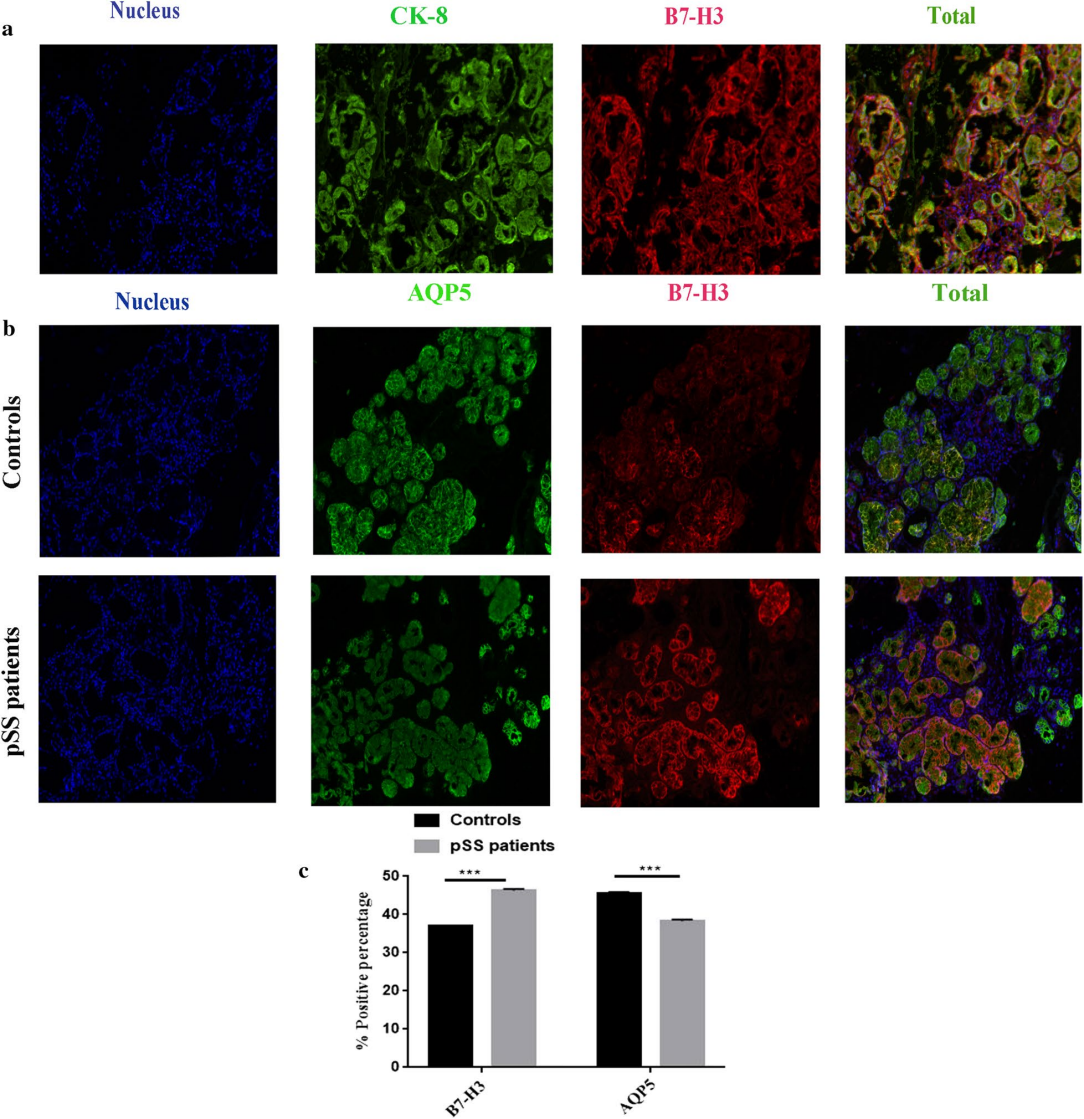
The location of B7-H3 expression were determined on the HSGE cells and the relationship between B7-H3 and AQP5. **a** The co-localization of B7-H3 and CK-8 in salivary gland confirmed that B7-H3 was on HSGE cells by immunofluorescence. **b** The expression of B7-H3 and AQP5 was determined by immunofluorescence in healthy and pSS salivary gland. **c** Quantitative analysis of immunofluorescence staining

The co-localization of CK-8 and B7-H3 was used to determine B7-H3 expression in epithelial cells (Fig. 3a). Previous research suggests that AQP5 contributes to salivary secretion in patients with SS [23]. Immunofluorescence of B7-H3 and AQP5 showed co-localization of these two proteins in the salivary gland (Fig. 3b, c). The positive ratio of B7-H3 or AQP5 in pSS patients’ and healthy controls’ salivary gland was 46.77% vs. 36.958% (B7-H3) or 38.77% vs. 45.75% (AQP5), respectively. The expression of AQP5 in pSS patients was significantly lower than that in healthy controls (*P *< 0.0001).

### The impact of B7-H3 expression on HSGE cells apoptosis

**Fig. 4:**
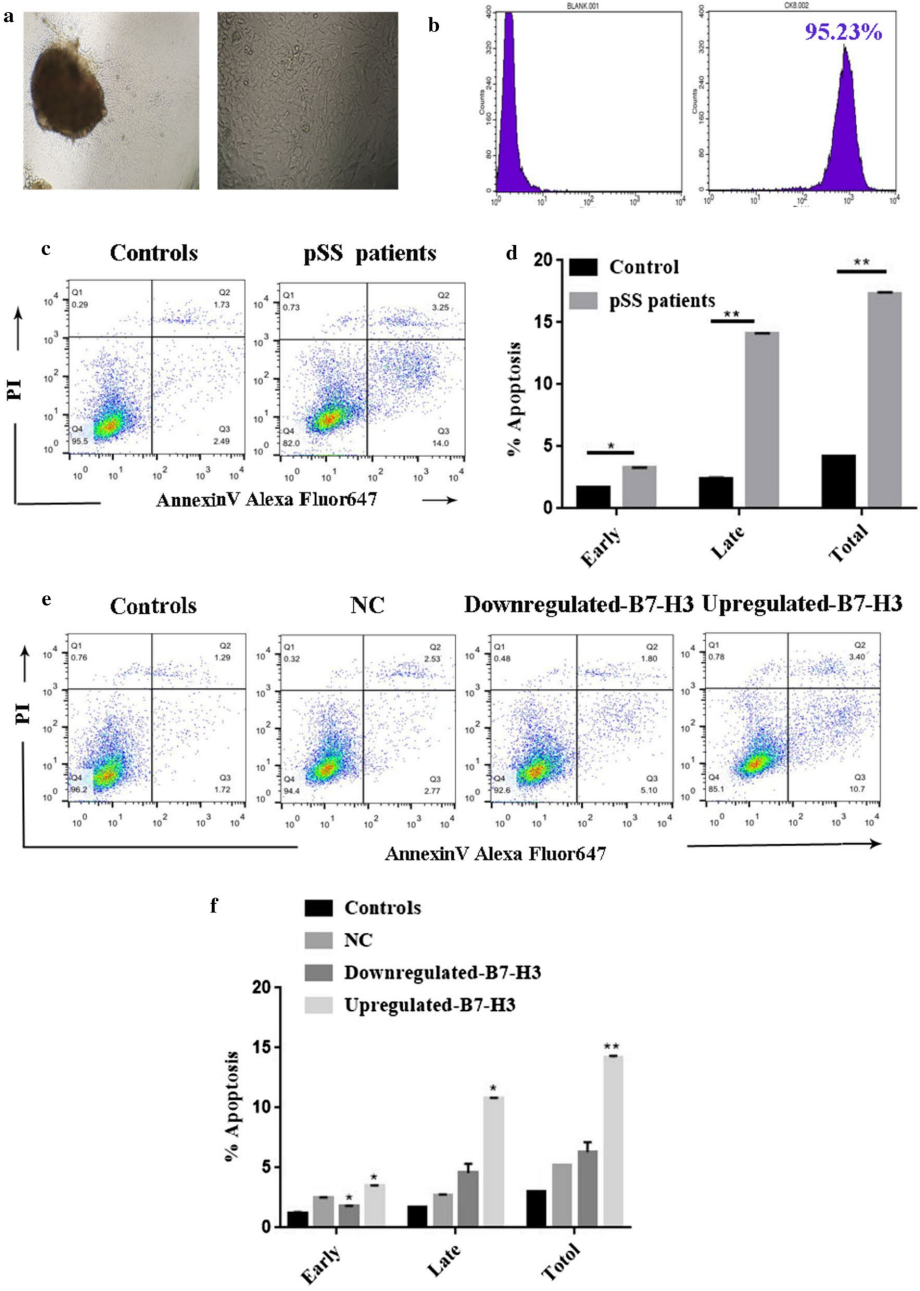
The impact of B7-H3 expression on HSGE cells apoptosis. **a** The epithelial characteristic of cultured HSGE cells was verified routinely by morphology. **b** HSGE cells labeled by CK-8 (95.23%) (right histogram) and blank control (left histogram). **c** By flow cytometry, we tested the effects of B7-H3 in primary HSGE cells on apoptosis. **d** The total apoptosis is the sum of early and late apoptosis. Quantitative results of the total percentages of apoptotic primary cells in the healthy control group and pSS patient group. *P < 0.05 vs. the healthy controls. **e** The cells were transfected for 48 h before detection. The apoptosis rates of control, upregulated-B7-H3 or downregulted-B7-H3, NC of HSGE cell lines were analyzed. **f** Quantitative results of the total percentages of apoptotic cells in the control, upregulated-B7-H3, downregulted-B7-H3, NC group. *P < 0.05 vs. the NC group

The epithelial characteristic of cultured primary HSGE cells was verified by morphology and the normal expression of epithelial-specific marker CK-8 (Fig. 4a, b). The main form of the cell line is similar to paving stone (Fig. 4a). CK-8 was localized in the cytoplasm of epithelial cells. CK-8 expression in salivary gland cells in vitro was tested by flow cytometry (Fig. 4b). From the results of flow cytometry, HSGE cells accounted for 95.23% of total primary cells. To determine the role of B7-H3 in primary HSGE cell apoptosis, we selected the primary cells from healthy controls and pSS patients. The results showed that the total apoptosis rate (the sum of early and late apoptosis) of primary HSGE cells in pSS patients was upregulated compared with healthy controls by Flow cytometry (*P *= 0.0065) (Fig. 4c, d). By knockdown and overexpression of B7-H3 in HSGE cells, we verified the results again. We measured cell apoptosis in the upregulated-B7-H3, downregulated-B7-H3, NC, control cell lines. The results of Annexin V/PI double staining showed that the total apoptosis rate of HSGE cell lines increased in the upregulated-B7-H3 group (*P *= 0.0071) while there were no alterations in downregulated-B7-H3 group(*P *= 0.3179) compared with NC group (Fig. 4e, f). The effect of upregulated-B7-H3 was consistent with that in primary cells in pSS patients.

### The impact of B7-H3 expression on HSGE cells proliferation

**Fig. 5:**
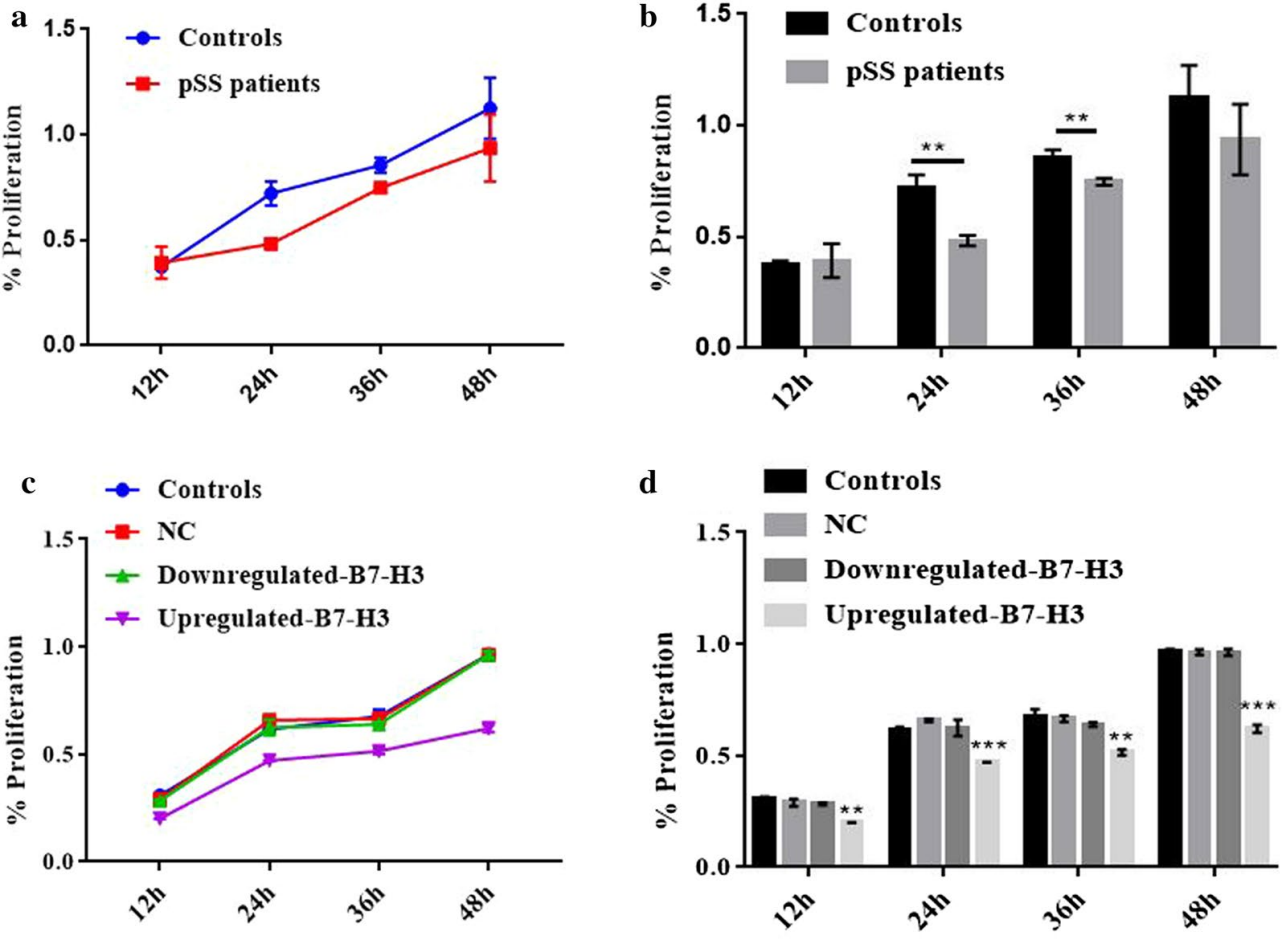
The impact of B7-H3 expression on HSGE cells proliferation. **a** Primary cell proliferation was detected by CCK-8. **b** Quantitative analysis of the cell proliferation. *P < 0.05 vs the healthy controls. **c** Cell proliferation of the control, upregulated-B7-H3, downregulated-B7-H3, NC group was detected. **d** Quantitative analysis of the transfected cell proliferation. *P < 0.05 vs the NC group

The effects of B7-H3 on HSGE primary cells proliferation were detected by CCK-8 assay. Compared with the healthy control group cells (Fig. 5a, b), the pSS patients group cells showed significant decreased cell proliferation rates at 24 and 36 h (all *P *< 0.05). In order to investigate the effect of B7-H3 on proliferation in HSGE cell lines (Fig. 5c, d), we knock down and overexpress B7-H3 in HSGE cells. The upregulated-B7-H3 group decreased cell proliferation more obviously at 12, 24, 36 and 48 h compared with the NC group (*P *< 0.05). However, there is no statistical significance in downregulated-B7-H3 group.

### The impact of B7-H3 on relative protein expression

**Fig. 6:**
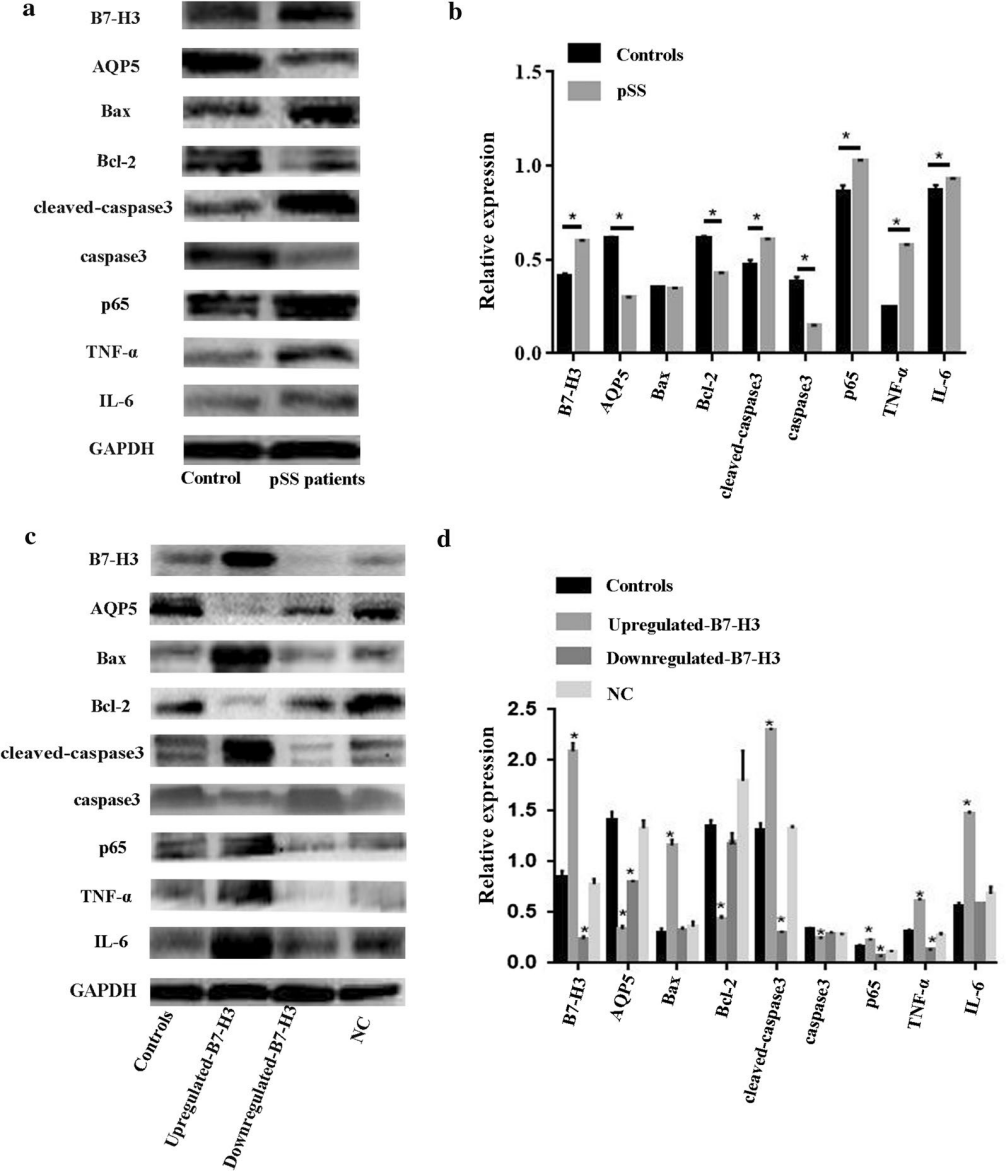
The impact of B7-H3 on the relative of protein expression. **a**, **b** We selected the primary cells from healthy controls and pSS patients. We compared the expression of B7-H3 protein and other molecules in primary HSGE cells by western blot analysis. *P < 0.05 vs the healthy controls. **c**, **d** The cells had been transfected for 48 h before detection. We compared the expression of B7-H3 protein and other molecules in downregulated-B7-H3, upregulated-B7-H3, NC, control group by western blot analysis. GAPDH as a positive control was probed. *P < 0.05 vs the NC group

Recent studies have shown that B7-H3 promoted metastasis and invasion of pancreatic cancer cells via the TLR4/NF-κB pathway [24]. p65 is an important transcription factor that plays a vital role in adaptive and innate immunity. In order to explore the relation between NF-κB pathway and the regulatory network of primary cell inflammation and apoptosis, we measured the expression levels of p65, the anti-apoptotic protein Bcl-2, the pro-apoptotic protein Bax, cleaved-caspase3, caspase3 and inflammatory cytokine IL-6, TNF-α (Fig. 6a). The result showed that p65 activity was higher in pSS patients (Fig. 6b). The expression of Bcl-2, caspase3, AQP5 was decreased and cleaved-caspase3, IL-6 and TNF-α was increased in comparison with those in the healthy controls (all *P *< 0.05). Those results indicated that the expression of B7-H3 promoted cell inflammation and induced cell apoptosis through NF-κB pathway probably. However, the expression of Bax had no difference between pSS patients and healthy controls. The cells had been transfected for 48 h before detection. Upregulated-B7-H3 and downregulated-B7-H3 were established in HSGE cell lines (Fig. 6c). Western blot assay showed that overexpression of B7-H3 significantly increased p65 expression (Fig. 6c, d). The expression of Bcl-2, AQP5, caspase3 was reduced and Bax, cleaved-caspase3, TNF-α, IL-6 were increased (all *P *< 0.05). However, the knockdown of B7-H3 only reduced p65, cleaved-caspase3, TNF-α expression (all *P *< 0.05). The expression of AQP5 was increased. However, there was no significant difference in the expression of Bax, Bcl-2 and IL-6. The results indicated that B7-H3 overexpression played a vital role and adjusted p65 activity in HSGE cell lines.

## Discussion

pSS is a systemic autoimmune disease with infiltration of periductal lymphocytes in saliva and lacrimal glands, which can result in down-regulated secretory function, dry mouth and eyes. It was reported that abnormal activation of CD4+ T cells and B cells have close relationship with development and pathogenesis of pSS.

B7 family members have recently drawn attention, especially following the success of anti-CTLA4 and anti-PD1/PDL1 therapy in advanced stage melanoma and other solid tumors [25, 26]. B7-H3, which belongs to the B7 superfamily, is a type I transmembrane protein originally detected in immune cells, such as dendritic cells and specific T cell subsets [27, 28], as well as in a variety of human solid tumor cells, including melanoma [29], breast cancer [30], ovarian cancer [31], pancreatic cancer [32], and gliomas [33, 34]. B7-H3-related studies in cancer patients have suggested a relationship with antitumor immune responses [34]. B7-H3 has been considered to be the third group of immune checkpoints [35]. However, the role and regulatory mechanism of B7-H3 in tumor immunity is controversial [36]. B7-H3 is thought to participate in the regulation of T cell-mediated immune response. Recent studies showed that B7-H3 had an inhibitory effect on immune regulation [37, 38]. However, recent papers showed that B7-H3 enhances T cell proliferation and cytotoxicity as well as IFN-γ, TNF-α and IL-10 production [27]. In SLE, B7-H3 expression was positively correlated with SLE disease activity index (SLEDAI) score and the disease activity index [39]. We suspect that B7-H3 molecule may act as a double-edged sword in the human body. B7-H3 has not been studied in pSS. In our study, the expression of B7-H3 in peripheral blood, salivary glands and saliva in pSS patients were higher compared with controls. B7-H3 expression in serum had positive correlation with the grade of the salivary glands. It was proposed that B7-H3 overexpression inhibited the activation and proliferation of T cells and sustained immune responses to autoantigens, and then affected multiple visceral organs and further aggravating the immunopathological damage caused by pSS. Those results firstly highlighted the effect of B7-H3 and the potential mechanism in pSS.

PD-1/B7-H1 is also viewed as a negative costimulatory molecule in the B7/CD28 superfamily. However, our results showed that the expression of PD-1/B7-H1 in peripheral blood, salivary glands and saliva in pSS patients were higher than that in healthy controls. The expression of B7-H1 in serum was positively correlated with the grade of the salivary glands. It was hypothesized that PD-1, which was abnormally elevated in pSS patients, may combine with B7-H1 in serum excessively and block the binding of PD-1 on the surface of T lymphocytes with B7-H1 on the surface of monocytes, glandular epithelial cells, and tissue cells. Therefore, the activated CD4+ T cells evaded the negative regulatory signals emitted by PD-1/B7-H1. The mechanism of PD-1/B7-H1 need to be further studied in pSS.

It was interesting that the expression of B7-H3 was different between healthy controls and pSS patients in saliva, but there are no differences on other relative factors. As we all known, saliva is an appropriate body fluid for biomarker detection because it is easy and noninvasive to collect and include diverse content of metabolites, proteins, and nucleic acids. Many of these molecules can be served as potential biomarkers for disease prognosis and diagnosis [40]. Saliva proteomics is a high-throughput analytical technique used to explore new candidate protein biomarkers and describe molecular processes associated with saliva secretion disorder in pSS. Previous study found that an abnormal distribution of AQP5 in HSGE cells might be associated with poor saliva secretion [23]. In addition, apoptosis-related molecules and inflammatory cytokines might influence the distribution of AQP5. Some studies had revealed that the high expression of AQP5 inhibited cell apoptosis and promoted cell proliferation [41]. B7-H3 expression showed negative correlation with AQP5 protein, positive correlation with apoptotic proteins and inflammatory cytokines in primary cells. We speculated that B7-H3 may affect the function of AQP5 by promoting epithelial cell apoptosis.

Previous studies also had shown that B7-H3 could participate in monocyte/macrophage-mediated inflammatory responses by amplifying p65, LPS and MAPK p38 signaling [42]. Additionally, B7-H3 promoted the invasion and metastasis of pancreatic carcinoma cells via the TLR4/NF-κB pathway. p65 activity is critical for immune system function and inappropriate p65 activation induce inflammatory responses and tumorigenesis. There was increasing evidence that dysregulation of p65 activity in glandular epithelial cells led to Sjögren’s-like features [17]. NF-κB is involved in the transcription of proinflammatory cytokines, such as tumor necrosis factor (TNF), IL-6, interleukin-1β (IL-1β), and monocyte chemoattractant protein 1 (MCP-1) [43]. Apoptosis of SG epithelial cells is also thought to be linked with autologous antibodies to SG epithelial cells exposed to pSS and increased levels of TNF-α activity [44]. The expression of TNF-α not only increased in the SG in pSS, but also played a key role in epithelial cells apoptosis and induces cellular damage [45]. Some researchers have showed that overexpression of TNF in mouse model reduced Bcl-2 expression and triggered the downstream caspase cascade, leading to intrinsic apoptotic pathway activation, that ultimately lead to adverse cardiac remodeling in the adult heart [46]. We suspected that B7-H3 increased cell inflammation, participated in cell apoptosis and decreased salivary secretion through NF-κB signaling pathway. To determine the relationship between B7-H3 and NF-κB signaling pathway, we evaluated the apoptosis and proliferation effect of B7-H3 in HSGE cell.

Our study revealed that B7-H3 could not only increase the activity of NF-κB pathway, the expression of TNF-α, IL-6, cleaved-caspase3 and Bax, but also decrease Bcl-2, caspase3, AQP5. We concluded B7-H3 could increase inflammation and induce cell apoptosis of HSGE cells via the NF-κB pathway. In short, B7-H3 might play a critical role in cell inflammation and apoptosis which may represent a potential mechanism of decreased salivary secretion in pSS patients.

## Conclusions

In summary, B7-H3 was overexpressed in pSS patients. Moreover, upregulation of B7-H3 could increase inflammation and induce apoptosis in HSG cell lines by regulating p65 activity. B7-H3 might be a promising target for pSS therapy.

## Data Availability

All data generated or analyzed during this study are included in this published article if additional information. If any additional information is required it may be obtained by request with the corresponding author.

## Abbreviations

AECG: American-European Consensus Group
C3: complement 3
C4: complement 4
CCK-8: Cell Counting Kit 8
ELISA: enzyme-linked immunosorbent assay
ESR: erythrocyte sedimentation rate
EULAR: European League Against Rheumatism
ESSDAI: EULAR primary Sjögren’s syndrome disease activity
ESSPRI: EULAR primary Sjögren’s syndrome patient-reported indexes
HSGE: human salivary gland epithelial gland
IHC: immunohistochemistry
NC: negative control
pSS: primary Sjögren’s syndrome
PMSF: phenylmethanesulfonyl fluoride
PI: propidium iodide
PD-L1/B7-H1: programmed death ligand 1
PD-1: programmed death 1
RA: rheumatoid arthritis
SLE: systemic lupus erythematosus
SLEDAI: SLE disease activity index
UWS: unstimulated whole saliva
WB: western blot
